# Antitumor Efficiency of Electrochemotherapy by High and Low Frequencies and Repetitive Therapy in the Treatment of Invasive Ductal Carcinoma in Balb/c Mice

**Published:** 2012-08-31

**Authors:** Zeinab Shankayi, Seyed Mohammad Firoozabadi

**Affiliations:** Department of Medical Physics, Faculty of Medical Sciences, Tarbiat Modares University, Tehran, Iran

**Keywords:** Electrochemotherapy, Low Pulse, High Pulse, Repetitive Treatment, Mice

## Abstract

**Objective:**

In electrochemotherapy (ECT), there is an unpleasant sensation of muscle contraction when using a low frequency (1 Hz). Therefore, by increasing the pulse frequency above the tetanic frequency this painful sensation can be reduced. The aim of the present study is to compare the treatment efficiencies of low and high frequency ECT, and estimate the effect of its repeated sessions.

**Materials and Methods:**

We transplanted invasive ductal carcinoma into the flanks of female Balb/c mice. ECT was performed on the mice by the use of 8 pulses, 1000 v/cm, of 100 µs duration at 1 Hz and 5 kHz repetition frequencies along with intra-tumoral injections of bleomycin. We also used this ECT protocol for the second therapy session six days after tumour regrowth. The effect of treatment was measured by calculating the tumor volumes for 24 days following treatment. Statistical analysis was performed with ANOVA.

**Results:**

ECT at 1 Hz and 5 kHz pulse frequencies demonstrated significant inhibition of tumor growth, but after the first treatment the tumours began to regrow. Repetitive ECT sessions increased the curability of tumors up to 40% in the group treated by 1 Hz frequency and 60% in the group treated with 5 kHz frequency.

**Conclusion:**

Our results demonstrate that the effects of 1 Hz and 5 kHz pulse repetition frequencies are comparable for inhibited tumour growth. Repetitive treatment can improve the effectiveness of ECT.

## Introduction

Electrochemotherapy (ECT) is a local, high efficiency treatment that combines chemotherapy agents with high voltage, short electric pulses. Electric pulses increase cellular permeability and improve the delivery of chemotherapy drugs such as cisplatin and bleomycin into the tumor cells. This new approach increases the local toxicity of these drugs, therefore decreasing the chemotherapy doses needed for therapy and their side effects (1-5).

ECT, with high voltage direct current electric field (1000-1300 v/cm) and a short duration (100 µs), significantly enhances the toxicity effect of bleomycin as a non-permeant therapeutic drug. Bleomycin causes single and double strain DNA breaks and kills tumor cells (4-8).

Preclinical and clinical studies have demonstrated the high antitumor efficiency of ECT. The advantages of this therapy are that it requires a low drug concentration, has good antitumor effectiveness, and reduces systemic toxicity (4, 5, 9).

In an ECT protocol, a train of 8 pulses at 1 Hz frequency, high voltage amplitude (1000-1300 v/cm), and 100 µs duration is needed for treatment. Low frequency and high amplitude pulses cause muscle contraction for each pulse (8 muscle contractions for 8 pulses), which creates an unpleasant sensation for patients (10-12). Therefore, researchers have attempted to prevent this painful sensation by using either high frequency or low amplitude electric pulses (10-16). In the high frequency approach for pain reduction), researchers have shown that by increasing the frequency of electric pulses above the tetanic frequency, it would be possible to decrease the number of contractions with increasing the uptake of non-permanent molecules (10, 11). This approach was performed at 5 kHz in an *in vivo* experiment and a similar antitumor efficacy was observed. The 5 kHz frequency ECT not only decreased the duration of therapy from 8 seconds to 1.6 ms, but also enabled the therapy to be tolerable for patients (11, 13). On the other hand, low amplitude ECT reduced pain intensity. Other researchers have shown that with a low amplitude and long duration, the electric pulse curable rate increased (14-16). In a hybrid approach, we have shown that the 5 kHz frequency combined with low electric pulses inhibited tumor growth and pain (17).

The first approach is already commercially available. The final two approaches have been examined *in vivo*, however do not yet have a standard protocol. Although successful results of 1 Hz and 5 kHz ECT have been seen, some treated tumors began to regrow after the first therapy session. This regrowth has been observed in all treatment protocols (17-19). We suggest the application of repetitive tumor treatment (20), however there is a lack of data that clearly shows the role of repetitive therapy in the treatment of tumors.

The aim of this study is to evaluate the efficiencies of high amplitude (1000 v/cm) ECT at low (1 Hz) and high (5 kHz) frequencies in invasive ductal carcinoma tumors, and to estimate the effect of repetitive therapy in these two ECT protocols.

## Materials and Methods

### Mice and tumors

In this experiment, inbred female Balb/c mice aged 6-8 weeks and that weighed 18-20 grams were purchased from Pasteur Institute, Tehran, Iran. They were maintained at 22℃ with a natural day/night light cycle for seven days for adaptation.

An invasive ductal carcinoma tumor was purchased from Pasteur Institute, Tehran, Iran. A 0.5 mm3 fragment of invasive ductal carcinoma was transplanted into the flank of each mouse. About two weeks after transplantation, when the tumor's largest diameter reached about 1 cm, the mice were randomly divided into treatment groups. The sample size was calculated by the formula: n= (z_1-α/2_+ z_1-β/2_)^2^ σ^2^/(µ_0_-µ_1_)^2^ where α=0.05, β= 0.20, z_1-α/2_=1.96, and z_1-β/2_=0.84. This research was approved by the Ethics Committee of Tarbiat Modares University.

### Tumor treatment

ECT was performed by injecting bleomycin (Nippon Kayaku Co. Ltd., Tokyo, Japan) directly into the tumors. Bleomycin was diluted in normal saline (1.5 mg/ml) after which 0.016 ml/g of this solution was injected into the tumors. Two minutes after the intra-tumoral injection of bleomycin, electric pulses were delivered. Electric pulses were applied to the tumors by an ECT-SBDC (designed and produced in the Small Development Center and Electromagnetic Laboratory of the Medical Physics Department of Tarbiat Modares University, Tehran, Iran). Eight 100 µs square-wave electric pulses of 1000 v/cm amplitude with a repetition frequency of 1 Hz and 5 kHz were delivered via two parallel stainless-steel electrodes placed subcutaneously on opposite sides of the tumors.

### Tumor volume monitoring

We monitored tumor volumes by measuring the diameters along the two largest dimensions with a digital caliper every three days (each diameter was measured three times). Tumor volumes were calculated using the formula V=πab^2^/6, where "a" is the larger diameter and "b" the smaller diameter. Tumor growth was normalized by the following: [the tumor volume measured at n days after treatment (Vn)/the tumor volume measured in the treatment day (V0)]×100. The inhibition rates of tumor growth were calculated according to the formula: inhibition rate (%)=[1-tumor volume (treatment group)/tumor volume (sham)]×100.

### Statistical analysis

Statistical analyses were performed using SPSS Inc. (Copyright 1993-2007, Polar Engineering and Consulting, Sept 13, 2007) for Windows 16.0. All data were tested for normality. One-way ANOVA was performed, followed by LSD, and statistical difference analyses by t test. P<0.05 was considered significant for rejection of the null hypothesis.

## Results

### Electrochemotherapy with 1 Hz and 5 kHz pulses

**Fig 1:**
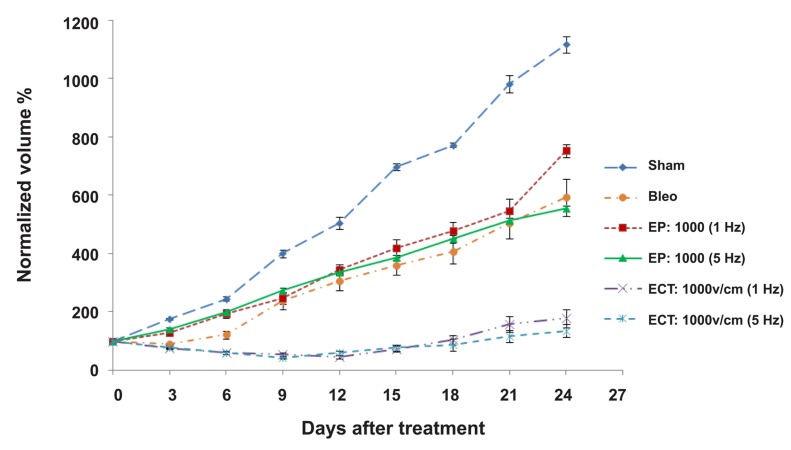
Electrochemotherapy (ECT) of tumors in mice at different frequencies. The results are presented as mean ± SE. EP; Electric pulses alone, ECT; Electrochemotherapy (BLM + EP).

**Fig 2:**
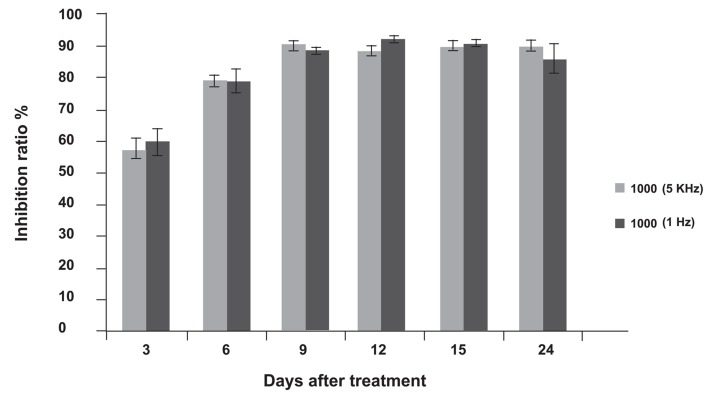
Electrochemotherapy (ECT) of tumors in mice at different frequencies. The results are presented as mean ± SE.

In this part of our study we estimated the effects of high and low frequencies in ECT. We compared two ECT protocols: 8 pulses, 1000 v/cm electric field strength, and 100 µs pulse duration at 1 Hz and 5 kHz pulse repetition frequencies. Both protocols significantly inhibited tumor growth (p<0.05; Fig 1). Our results showed that both 1 Hz and 5 kHz pulses reduced tumor volumes; however tumors began to grow again after the first session. For 24 days post-treatment, tumor volumes in both ECT groups were not significantly different (p>0.05). Electric pulses alone had a significant effect in inhibiting tumor growth (p>0.05; Figs. 1, 2).

**Table 1:** Antitumor efficiency of electrochemotherapy (ECT) with different pulse repetition frequencies and repeated treatment


Therapy	Sham	EP 1 Hz	EP 5 kHz	ECT 1 Hz (Once)	ECT 5 Hz (Once)	ECT 1 Hz (Twice)	ECT 5 Hz (Twice)

N	8	8	8	10	10	5	5
PR	0	0	0	6	6	3	2
CR	0	0	0	0	0	2	3


EP; Electric pulses alone, ECT; Electrochemotherapy (BLM + EP), N; Number of animals in each experimental group, CR; Complete response and PR; Partial response.

### The effect of repetitive electrochemotherapy treatment

Our results showed that tumors in the ECT group grew. In order to achieve sufficient results, we repeated ECT six days after the tumors began to grow, in the same manner as the first session therapy for each group. Complete response was not observed in groups with one ECT session. With repeated ECT there were two mice in the 1 Hz group and three mice in the 5 kHz ECT group that had complete responses (total disappearance of the tumor). The tumors with partial response (at least 50% decrease in tumor size) did not grow (Table 1).

## Discussion

In the first step of the present study, we verified ECT by two different frequencies (1 Hz and 5 kHz) in the treatment of invasive ductal carcinoma. Our results showed that these two conditions affected tumor growth in a similar way for the first 24 days after treatment (Fig 1).

Preclinical and clinical studies have demonstrated high antitumor efficiency of standard ECT (1300 v/cm, 8 electric pulses by 100 µs duration and 1 Hz frequency pulses). Although advantageous, the most unpleasant side effect of ECT is muscle contractions related to sensations during pulse delivery (10-12, 18). Miklavcic and co-workers (11) have demonstrated that it would be possible to reduce these unpleasant sensations and painful effects by using pulse repetition frequencies higher than the tetanic frequency as an alternative to the standard pulse frequency of 1 Hz.

An important part of ECT is that it facilitates the uptake of non-permeable molecules to cells. In another study, the uptake of non-permeable molecules at the highest repetition of frequencies has been examined. This *in vitro* study has shown that the uptakes have stayed at similar levels as the uptake at 1 Hz but different frequencies in different voltages corresponded to the maximum uptake value (10).

Recently, clinical experiments did not show a difference in the efficiency of ECT by 1 Hz or 5 kHz repetition frequencies of electric pulses, but therapy at a frequency of 5 kHz reduced pain in patients (13, 19). In addition to increasing cellular permeability, the electrical pulse also reduced blood flow in tissues (18, 21-25). By decreasing tumor blood flow, drugs become trapped in the tumor, which provides a longer time for the drug to act. This modification in blood flow could be particularly beneficial for intra-tumoral drug administration, as this would decrease drug wash out from the tumor (25). Sersa et al. have examined the effects of ECT using 1 Hz and 5 kHz in the treatment of mouse sarcoma. Their study has concluded that ECT by these two protocols were effective as treatments, however in contrast to our results, they have shown that 5 kHz electric pulses were less effective than 1 Hz pulses (26). They also reported that the application of 5 kHz frequency in tumors caused an immediate reduction in blood perfusion, comparable with 1 Hz electric pulses. However tumor perfusion recovery was faster in the group treated with a frequency of 5 kHz. The researchers concluded that electric pulses with a 5 kHz frequency had less effect on blood perfusion of mouse sarcoma tumors (26). Recently, Raeisi showed that a 5 kHz pulse repetition frequency had a comparable effect with 1 Hz frequency on blood perfusion of invasive ductal carcinoma, but high frequencies induced a more intensive effect on blood perfusion reduction in the short time after delivery of the electric pulses (27).

This contrast led us to verify the effect of these two protocols in the treatment of invasive ductal carcinoma tumors (the same tumors and conditions as the Raeisi et al. experiment) and we observed that 5 kHz electric pulses were more effective than 1 Hz pulses. Our results with the same ECT protocols as Sersa et al. (26) and the same tumors and treatment conditions as Raeisi et al. (27) have predicted a variation in the reduction of blood perfusion in different tumors, causes to obtain different treatment efficiency Therefore, as the membrane electropermeabilization facilitates drug transport and its accumulation in tumor cells, the change in tumor blood flow is involved in the antitumor effects of ECT. The combination of these two mechanisms can inhibit tumor growth (21-26). It is possible that different tumors need different ECT protocols and our results imply that ECT protocol effectiveness depends on the tumor types.

In the present study we attempted to improve the effect of ECT with repetitive therapy sessions. Initially we observed growth in all tumors. After six days of tumors regrowth, ECT in the same manner as the first session was repeated.

Researchers have suggested three different approaches to improve ECT efficiency: i.changing the electrode or electric field orientation which leads to improved electric field distribution, ii. intra-tumoral injection of chemotherapy drugs which can cause an increase in the aggregation of the drug and iii. repetitive treatment of tumors that regrow after one therapy session which could create better tumor control (18).

We have examined the third approach and demonstrated that ECT effectiveness increased with multiple treatments (20). In ECT, the tumor volume that is covered by induced electric pulses is important, as larger tumors that cannot be covered by an electric field in one session must be retreated. In these cases, repetitive ECT is very effective and treatment can be repeated three to six weeks after the first session (19, 28, 29). But our results, using repetitive treatment in tumors that are resistant to ECT have demonstrated that with repetitive treatment and choose suitable protocol could improve effectiveness of ECT in the treatment of tumors that were resistant to it (Table 1). We suggest repetitive treatment for tumors resistant to ECT, but more preclinical studies are needed to confirm our results.

## Conclusion

We have shown that the effects of 1 Hz and 5 kHz repetition frequencies are comparable in the treatment of invasive ductal carcinoma. Repetitive ECT is effective for tumors that resume growth after single session therapy in that it can control these types of tumors.
